# Metabolic dyshomeostasis by organophosphate insecticides: insights from experimental and human studies

**DOI:** 10.17179/excli2019-1492

**Published:** 2019-07-05

**Authors:** Apurva Kumar Ramesh Joshi, Bindhu Omana Sukumaran

**Affiliations:** 1 Department of Biochemistry, School of Sciences, Jain University, Bangalore, Karnataka, India 560041

## ⁯

***Dear Editor,***

Organophosphate insecticides (OPI), derived from phosphoric, phosphonic or phosphinic acids, find application as agents for controlling insect pest populations. OPIs elicit their characteristic toxicity by phosphorylating and inhibiting the enzyme, acetylcholinesterase (AChE). Cholinergic stress resulting from overstimulation of nicotinic- and muscarinic acetylcholine receptors is the chief mechanism of acute toxicity of OPI (Fukuto, 1990[9]; Sogorb and Vilanova, 2002[31]; Abou-Donia, 2003[2]; Costa, 2006[7]). The ubiquitous nature of AChE and its conserved physiological role in the regulation of neurotransmission means that non-target animals (including humans) are at risk of adverse outcomes in the event of exposure to OPI. Neurotoxicity, characterized by cholinergic and non-cholinergic outcomes, is the most studied aspect of OPI toxicity. However, it is now unequivocally recognized that the toxicity of OPIs goes beyond the realm of neurotoxicity.

A large number of animal studies explicitly demonstrate that OPIs have the propensity to cause hyperglycemia, perturbations in carbohydrate metabolism and endocrine dysregulations. Repeated exposure to OPI precipitates insulin resistance (studies listed in Table 1(Tab. 1); References in Table 1: Abdollahi et al., 2004[1]; Acker and Nogueira, 2012[3]; Fuentes-Delgado et al., 2018[8]; Hamza et al., 2014[11]; Joshi and Rajini, 2009[14]; Joshi and Rajini, 2012[15]; Joshi et al., 2012[13]; Kamath et al., 2008[17]; Lasram et al., 2008[18]; Lasram et al., 2015[19]; Liang et al., 2019[20]; Mostafalou et al., 2012[24]; Nagaraju and Rajini, 2016[26]; Nagaraju et al., 2015[25]; Ribeiro et al., 2016[29]; Salek-Maghsoudi et al., 2019[30]; Velmurugan et al., 2013[34]; Velmurugan et al., 2017[33]; Yousefizadeh et al., 2019[36]), a key component of metabolic syndrome. Extrapolating the outcomes of animal experimentation to the human situation is a challenging task. Experimental studies often employ doses much higher than doses of environmental relevance. However, several studies clearly (listed in Table 2(Tab. 2); References in Table 2: Amanvermez et al., 2010[4]; Ather et al., 2008[5]; Çolak et al., 2014[6]; Gifford et al., 2019[10]; Hui, 1983[12]; Montgomery et al., 2008[21]; Moon et al., 2016[22]; Moore and James, 1981[23]; Raafat et al., 2012[27]; Rao and Raju, 2016[28]; Sudhir et al., 2013[32]; Velmurugan et al., 2017[33]; Weizman and Sofer, 1992[35]; Yurumez et al., 2007[37]) establish that OPI exposure elicits metabolic dyshomeostasis in human subjects. A recent study demonstrates that the incidence of diabetes among Thai farmers positively correlates with OPIs such as chlorpyrifos, dicrotophos, dichlorvos, ethyl-p-nitrophenyl, mevinphos, monocrotophos and methamidophos (Juntarawijit and Juntarawijit, 2018[16]). Thus, it is evident that OPI exposure is a clear risk factor for metabolic dysregulations among those who are occupationally exposed. One has to take cognizance of the fact that levels of exposure to OPI among occupationally exposed people are likely to be higher than the general population. However, a recent study reveals that glycated hemoglobin levels correlate with plasma levels of OPI (due to environmental exposure), but not with AChE activity (Velmurugan et al., 2017[33]). This indicates that low-level OPI exposure may cause metabolic dysregulations. Hence, we believe that further studies are needed to evaluate the effects of low-level, chronic non-occupational exposure to OPI on metabolic health.

## Acknowledgements

Authors are thankful to Jain University, Bangalore for the support.

## Conflict of interest

The authors declare no conflict of interest.

## Figures and Tables

**Table 1:**
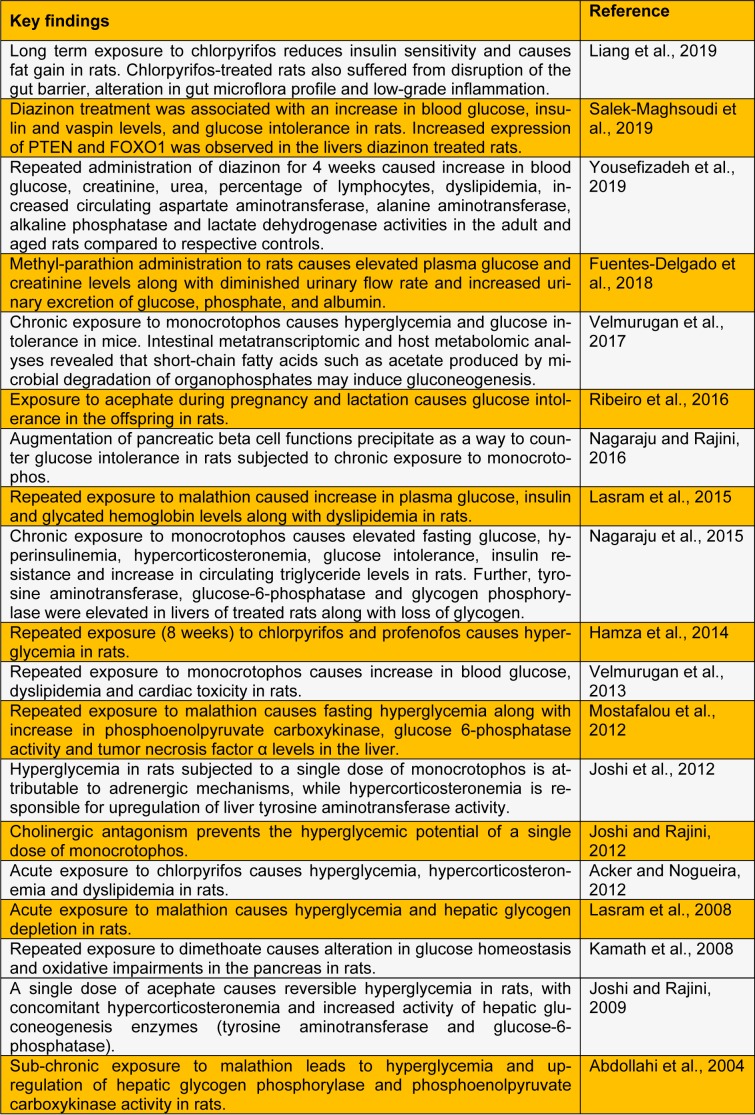
Experimental studies reporting metabolic dysregulations caused by organophosphate insecticides in rodent models

**Table 2:**
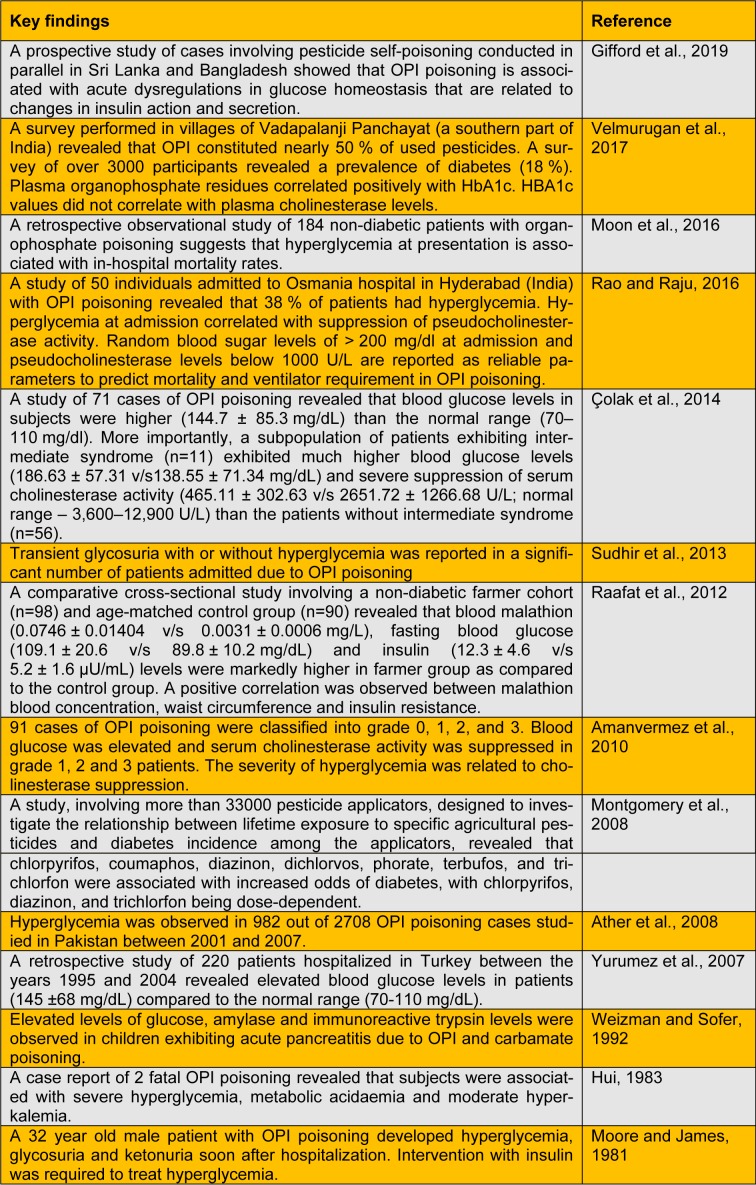
Studies that demonstrate the link between exposure to organophosphate insecticides and metabolic dysregulations in human subjects
